# Curcumin as a novel therapeutic candidate for cancer: can this natural compound revolutionize cancer treatment?

**DOI:** 10.3389/fonc.2024.1438040

**Published:** 2024-10-23

**Authors:** Shadiya Fawzul Ameer, Muna Yusuf Mohamed, Qubaa Ahmed Elzubair, Elham Abdullatif M. Sharif, Wisam Nabeel Ibrahim

**Affiliations:** Department of Biomedical Sciences, College of Health Sciences, QU Health, Qatar University, Doha, Qatar

**Keywords:** curcumin, cancer, chemotherapy, signalling pathways, nanotechnology

## Abstract

Cancer remains one of the leading causes of death worldwide. Despite advances in medical treatments, current therapeutic strategies, including radiotherapy, chemotherapy, targeted therapy, and surgical resection, have not significantly reduced the global incidence and mortality rates of cancer. Oncologists face considerable challenges in devising effective treatment plans due to the adverse side effects associated with standard therapies. Therefore, there is an urgent need for more effective and well-tolerated cancer treatments. Curcumin, a naturally occurring compound, has garnered significant attention for its diverse biological properties. Both preclinical studies and clinical trials have highlighted curcumin’s potential in cancer treatment, demonstrating its ability to inhibit the proliferation of various cancer cell types through multiple cellular and molecular pathways. This paper examines the antineoplastic properties, and the therapeutic mechanisms including cell signalling pathways targeted by curcumin that are implicated in cancer development and explores the challenges in advancing curcumin as a viable anticancer therapy.

## Introduction

Cancer is considered an emerging public health challenge worldwide in the 21st century. After cardiovascular diseases, cancer is the second most life-threatening disease. In 2020, there were nearly 10 million cancer deaths and almost 18.1 million new cancer cases globally (1–3). The high death rate is caused by the absence of effective cancer screening techniques and the mild or nonexistent symptoms that are seen in the early stages. As a result, most patients are diagnosed at an advanced stage (4, 5). At present, the overall efficacy of cancer treatment has not significantly increased despite advances in modern science and medical technology. As a result, multiple studies have predicted that there would be an increase in the incidence and mortality of cancer patients. For Instance, the survey conducted by Jianhui and his colleagues documented that by 2030, there will be a considerable increase of 31% in the global rates of cancer incidence and a 21% increase in mortality, respectively (6). In addition, the study conducted by Sung et al. stated that by 2040 cancer is anticipated to rise by 47% (1). Therefore, alternative strategies are an urgent need to improve cancer survival rates

Recent years have seen a considerable increase in the use of herbal medicine in scientific academies for the treatment of numerous diseases, including cancer. Several natural products—such as fruits, vegetables, tea, and spices—have garnered significant interest from researchers due to their potential for managing and preventing cancer. One of the many useful components of medicinal herbs is curcumin, which comes from the turmeric plant, scientifically known as “*longa Curcuma*.” *(* 7). Curcumin is one of the ingredients of curry spice and is widely utilized in the food industry. In a published study from 1985, the anti-tumor properties of curcumin were first shown (8). After that, many studies investigating the effects of curcumin in human cell lines and animal models of different carcinomas have been carried out (9). According to Aminudin et al., 2023, this turmeric-derived phytochemical compound offers various biological and therapeutic potentials (10). Additionally, regardless of the dose used, curcumin has demonstrated safety in medicine with minimal side effects (11). Despite this, curcumin has certain pharmacological drawbacks, including limited bioavailability and water solubility, which may make it less useful in clinical settings. Therefore, studies have suggested several approaches to overcome these challenges, including the use of curcumin derivatives, analogs, or nano-based drug delivery systems (12–14). This review aims to elucidate the anticancer properties of curcumin and its derivatives, analogs, and nano-based formulations in cancer treatment, focusing on their underlying mechanism of action via various signaling pathways. This endeavor will entail aggregating both experimental and clinical data concerning the mechanism of action of curcumin, with emphasis on the period from 2014 to 2024, a span chosen due to the discernible surge in curcumin’s utilization in the past decade

## Background

Curcumin, the golden spice in Asian countries is made from the rhizome of the turmeric plant *Curcuma longa*, a member of the ginger family*, Zingiberaceae*, with a molecular weight of 368g/mol and a melting point of 183°C (15–17). It is a polyphenolic molecule chemically, having two aromatic rings with one hydroxy and one methoxy substituent in each. The rings are joined by a seven-carbon chain that has two α-β unsaturated carbonyl groups (21, 22). This spice has been used for centuries in traditional medicine to treat various conditions, including cancer, skin disorders, diabetes, and inflammation. It increases blood circulation, eliminates stagnation, and decreases depression (18–20). The three main ingredients of commercially available curcumin are diferuloylmethane, which makes up 82% of the compound and is the most active; its derivatives, desmethoxycurcumin (15%) and bisdemethoxycurcumin (3%), are together known as “curcuminoids.” (1)

**Figure 1:**
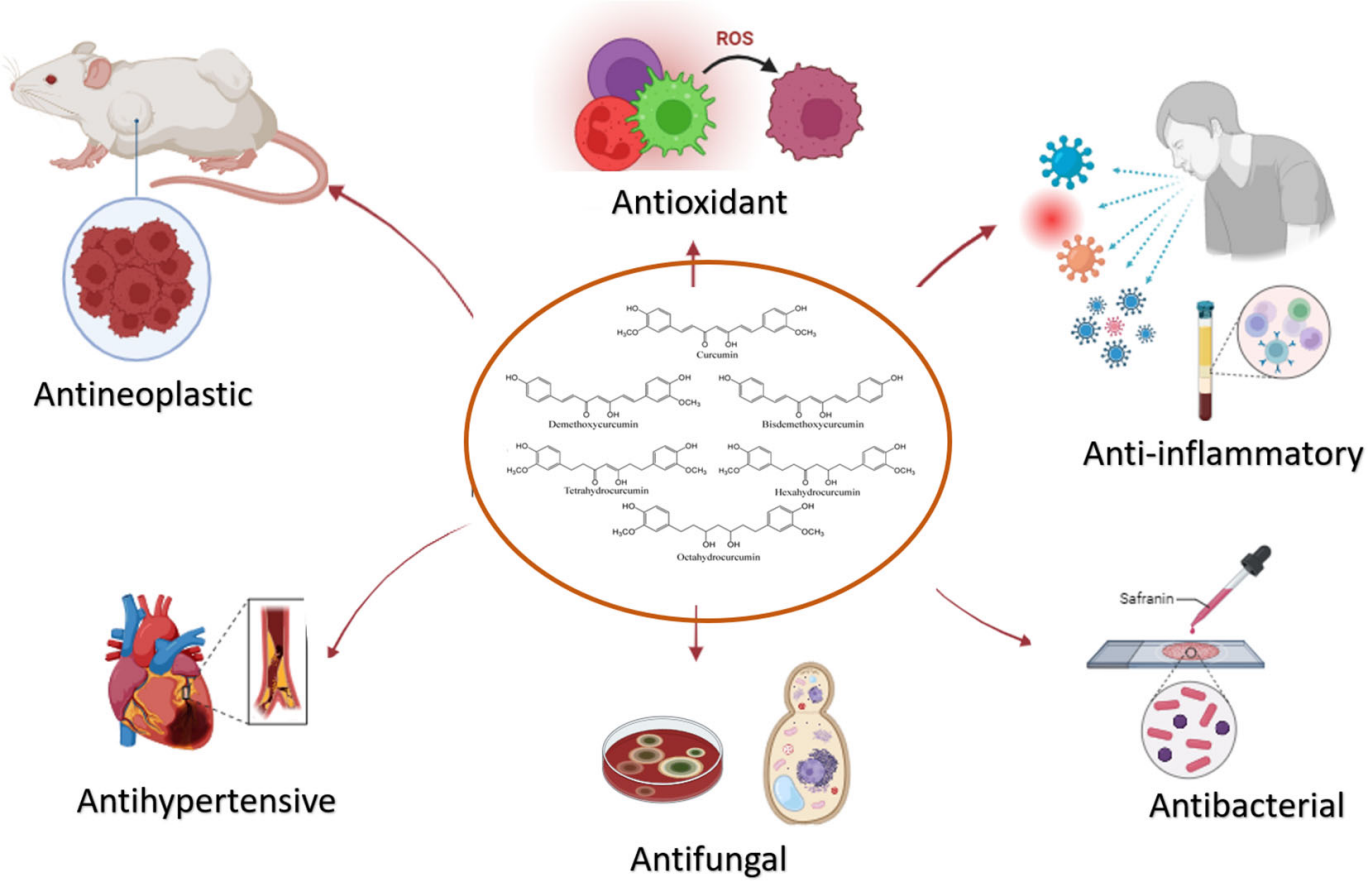
The potential therapeutic properties of Curcumin. Its multifaceted effects, encompassing anti-inflammatory, antioxidant, and antihypertensive properties, underscore its versatility in disease management.

The curcuminoids, which are primarily responsible for the pharmacological activity of turmeric, have been shown to have many advantageous characteristics, such as anti-inflammatory, antioxidant, and chemotherapeutic activities (23–25), illustrated in **Figure 1**. The anti-cancer properties of curcuminoids, such as angiogenesis, invasion, migration, metastasis, and cell survival and proliferation, are modulated through signaling pathways (26). This has been observed in multiple *in vitro* and *in vivo* research, for instance, the study conducted by Mengjie et al., observed that has shown that curcumin inhibits the metastasis and invasion of breast cancer via Hedgehog/Gli pathways by downregulating the genes related to the Hedgehog pathway (30). Although curcuminoids possess high anti-inflammatory and anti-tumor properties, their low level of hydrogenation, high level of methoxylation, and high level of unsaturation of the diketone moiety have resulted in curcuminoids to be an unstable drug to implemented into clinical trials (2), since, the poor solubility of curcuminoids and short biological half-life results in rapid metabolism and elimination by the liver. Hence multiple strategies have been mitigated to enhance the bioavailability and pharmacodynamics of curcumin and implement it as a safe anti-cancer drug (3).

### Signaling pathways linked to curcumin for combating the neoplastic effects of cancer

**Figure 2:**
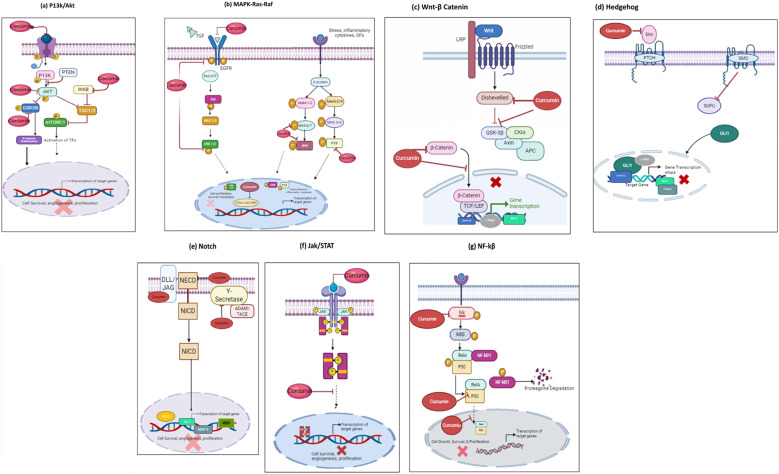
Mode of curcumin actions as an anti-cancer agent on the key molecular targets as an anticancer therapeutic Agent. Curcumin possesses anti-cancer properties by inhibiting signaling pathways and their downstream molecular targets; **(A)** P13k/Akt/mTOR pathway;**(B)** Ras/Raf/ERK pathway; **(C)** JAK1,2/STAT3 pathway; **(D)** Wnt/β-Catenin pathway; **(E)** NF-kβ pathway; **(F)** Hedgehog pathway; **(G)** Notch pathway. Molecular targets and signaling pathways that are downregulated by curcumin are noted by using ┤, respectively.

Curcumin, has gained significant attention for its anticancer properties, primarily due to its ability to modulate a wide range of cellular signaling pathways involved in cancer progression. These signaling pathways play critical roles in regulating key processes such as cell proliferation, apoptosis, angiogenesis, invasion, and metastasis, which are hallmarks of cancer. Curcumin’s unique ability to interact with multiple molecular targets allows it to disrupt these pathways, thereby exerting its anticancer effects. By inhibiting pro-survival and pro-inflammatory signaling cascades such as PI3K/Akt/mTOR, MAPK, Wnt/β-catenin, NF-κB, Hedgehog, Notch, and JAK/STAT3, curcumin effectively impedes cancer cell growth and promotes apoptosis. Understanding these pathways is crucial as it provides insights into curcumin’s multifaceted role in combating cancer, highlighting its potential as a powerful natural therapeutic agent that targets the molecular underpinnings of neoplastic diseases. In the coming sections curcumins interference with each signaling pathway will be explained and demonstrated in **Figure 2.**

### PI3K-Akt-mTOR pathway

Numerous biological processes, including cell division, growth regulation, metabolism, and cell survival, depend on the phosphoinositide 3-kinase (PI3K)/Akt and mammalian target of rapamycin (mTOR) signaling pathway. Overactivation of this signaling system is linked to metastasis, evasion of apoptosis, and cell proliferation which are all cancer hallmarks (6, 7). The AKT oncogene, the inactivation of the tumor suppressor gene PTEN, and the overexpression of growth factors like VEGF-A and EGF are specifically the main causes of the dysregulation of the PI3K/Akt pathway (8, 9). Research findings indicate that curcumin and its analogs have direct interactions with many molecules involved in this pathway, such as mTORC1 (mammalian target of rapamycin complex 1), AKT, and PI3K (10, 11). Curcumin further interacts with IκB kinase B (IKKβ) to target the PI3K/Akt pathway indirectly. Specifically, curcumin has been shown to decrease tumor growth by blocking IKKβ, an upstream regulator of mTORC1 (12). In addition, tyrosine phosphatase PTEN is the protein that negatively regulates PI3K/AKT activity through inhibiting of PI3K. AKT activation can result from PTEN gene mutation and methylation leading to cancer progression (124). In gastric cancer cell lines, curcumin increased the expression of PTEN protein, markedly reduced proliferation, and triggered apoptosis (13). Moreover, Glycogen Synthase Kinase-3 Beta (GSK3β) controls cell proliferation by phosphorylating proteins including cyclin D1, which affects the cell cycle. A typical feature of cancer is excessive cell proliferation, which can result from aberrant regulation of GSK3β. Negative regulation of GSK3β is caused by the PI3K/AKT pathway, which is frequently overactive in cancer. Increased cell survival and proliferation result from GSK3β inhibition caused by AKT-mediated phosphorylation. A crucial element of the Wnt signaling pathway, β-catenin, is regulated by GSK3β. In the absence of Wnt signaling, β-catenin, adenomatous polyposis coli (APC), and active GSK3β combine to form a complex with the Axin protein, which phosphorylates and degrades β-catenin (14). Curcumin directly interferes with GSK-3β activity, which decreases the level of β-catenin in the nucleus, in turn, prevents gene transcription (1). Additionally, a study conducted by Muhanmode et al., observed that curcumin in combination with resveratrol inhibits chemoresistance in epithelial ovarian cancer cells by enhancing the P13K pathway stimulating the Bax gene and inhibiting the Bcl_2_ gene thus inducing apoptosis (15). Furthermore, curcumin possess the ability to modulate the core pathways involved in glioblastoma (GBM) and induce cell cycle arrest, autophagy and paraptosis by suppressing the phosphorylation of Akt protein on Ser 473 thus resulting a decrease in tumor size in C6-implated Wistar rats (16).

### MAPK-RAS-RAF-MEK-ERK pathway

The Ras-Raf-MEK-ERK pathway, also known as the extracellular signal-regulated kinase (ERK) pathway or mitogen-activated protein kinase pathway (MAPK), is a transduction cascade that carries extracellular signals into the nucleus of cells, where specific genes are triggered for cellular survival, proliferation, and differentiation (17). Curcumin’s ability to modulate the MAPK-RAS-RAF-MEK-ERK pathway represents a promising mechanism for its anticancer activity by targeting multiple components of this signaling cascade, curcumin disrupts oncogenic signaling, promotes apoptosis, and inhibits cancer cell proliferation, highlighting its potential as a therapeutic agent in cancer treatment. As previously indicated, one of curcumin’s anticancer characteristics is its capacity to cause cancerous cells to undergo apoptosis by downregulating the MAPK pathways. For instance, curcumin activated the JNK and p38 MAPK pathways, which decreased the survival of retinoblastoma cells and caused Y79 cells to undergo apoptosis (18). Another study employed curcumin to activate the Ras-Raf-MEK-ERK pathways. The investigation demonstrated that curcumin elicited a downregulation of EZH2 expression via the phosphorylation and subsequent activation of ERK, JNK, and p38 in MDA-MB-435 cells. Furthermore, in MDA-MB-231 cells subjected to curcumin treatment, a discernible induction of apoptosis and autophagy was observed, correlated with heightened activity of ERK, JNK, and Beclin1. Guo et al., 2018, demonstrated that curcumin induces autophagy *in vitro* in acute lymphoblastic leukemia (Ph+ALL) cells. Their study provides evidence that curcumin does cause autophagy to occur in SUP B15 cells at precisely 4 and 8 hours following curcumin administration (19). In addition, a strong correlation has been seen between the regulation of the MAPK activity pathway and the invasion and metastasis of cancer by transforming growth factor beta 1 (TGF-β1), EGF, and its related receptor, EGFR. It was observed that curcumin inhibited EGFR phosphorylation driven by EGF, downregulated TGF-β2 production, and suppressed ERK activity in MDA-MB-468 cells (20). Furthermore, polyphenolic compounds in curcumin are studied to play a vital role as a treatment strategy for colorectal cancer as it has an anti-tumor effect to MEK inhibitor U0126 (21). Additionally, curcumin suppresses ERK and JNK signaling pathways by upregulating TFPI-2, which results in the inhibition of EMT and resulting the inhibition of pancreatic cell invasion and migration (22).

### Wnt/β-catenin pathway

Wnt/β-catenin signaling maintains and induces stem cell differentiation, which is involved in adipogenesis, hematopoiesis, and skin development. To activate Wnt/β-catenin signaling, β-catenin’s stability is crucial as it is the central constituent of the cadherin complex (17). Genes activated by Wnt pathway are transcribed by the transcription complex which will start the transcription of associated genes including cyclin D1, c-Myc, and MMPs (23, 24). A disruption in this process results in the build-up of β-catenin within the nucleus and amplifies the expression of several oncogenes, such as cyclin D1 and c-myc. Consequently, the Wnt/β-catenin pathway may potentially be a therapeutic target as it contributes to the development of cancer (1, 25). In a study, curcumin suppressed the proliferation of gastric cancer (GC) cells by downregulating the target genes of the Wnt/β-catenin pathway, namely Wnt3a, LRP6, β-catenin, c-myc, and surviving (26). Additionally, curcumin can significantly decrease the expression levels of β-catenin, cyclin D1, and c-Myc, and hinder the development of cancer cells via the Wnt/β-catenin pathway. For example, a study conducted by Wang et al., 2018 showed that curcumin inhibits lung cancer cell proliferation via the Wnt/β-catenin pathway. Their study found that curcumin inhibited the expression of β-catenin, as well as the expression of downstream cyclin D1 and c-Myc (27). Also, curcumin inhibited the proliferation of malignant cells in breast cancer cell lines (MCF-7 and MDA-MB-231) by blocking numerous sites along this route, which resulted in the reduction of disheveled, GSK3β, β-catenin, and cyclin-D1 (28). Furthermore, curcumin inhibited axin recruitment to the cell membrane and reduced β-catenin activity ultimately decreasing activation of the β-catenin target genes causing apoptosis in a human hepatocellular carcinoma cell line (29) Additionally, in the study conducted by Jian-Huang et al., they effectively abolished lung CSC traits as it reduced tumorshere formation and decreased Wnt/β-catenin and sonic Hedgehog pathways (4). Moreover, Hongling et al., demonstrated that curcumin can suppress osteogenesis via the upregulation of miR-126a-3p expression directly inhibiting LRP6 to block WNT activation. The research suggested that using curcumin as an anti-tumor agent may decrease bone mass (30). Furthermore, Zhu et al., demonstrated that curcumin inhibits EMT, cell invasion, and cell growth in hepatocellular carcinoma cell lines HepG2 and Huh-7, via the Wnt/β-catenin signal pathway (31, 32). Additionally, curcumin acts as an agonist of PPARϒ in the WNT signaling pathway (1).

### Hedgehog signaling pathway

Hedgehog (Hh) signaling pathways are essential for tissue homeostasis in adult organisms and embryonic development (33). Research has demonstrated that Notch and Hh signaling work together to promote cell development in several body systems (34). The primary elements of the Hh cascade are: Hh ligands including Indian (Ihh), Sonic (Shh), and Desert Hedgehog (Dhh), two transmembrane proteins [Patched (PTCH)] and Smoothened (SMO) and GLI transcription factors (GLI1, GLI2, and GLI3) (35). Dysregulations in Hh signaling lead to a variety of prenatal disorders, oncological cancers, and immunological abnormalities (33). Hh signaling cascade is initiated when the cell-surface receptor PTCH, which typically inhibits the activity of SMO, binds to the Hh ligand. Then, PTCH1 loses its inhibitory hold on SMO, causing GLI to accumulate nuclearly and target genes to become activated. These events encourage several carcinogenic traits in tumor cells (36). The Suppressor of Fused (SUFU) protein controls GLI activity in the absence of binding by blocking its translocation from the cytoplasm to the nucleus (37). The study conducted by Zhang et al., observed that curcumin suppressed the expression of Shh, Gli1 and Foxm1in SGC-7901 cells both in mRNA and Protein, thus resulting in cellular migration, invasion and cytoskeletal remodelling ability were decreased and cells were arrested in S phase of the cell cycle (38). In a study, glioma cells were exposed to curcumin for varying lengths of time to see if it had any effect on the Hh pathway. They observed that curcumin decreases GLI1 levels intracellularly and intranuclearly in glioma cell lines. Additionally, they demonstrated that curcumin might promote glioma cell apoptosis by causing a significant decreases in the expression of Bcl-2, an anti-apoptotic effector and direct target of Hh signaling (39). This finding aligns with another study that showed curcumin promoted Bcl‐2 and apoptosis in glioma cells (40). Additionally, Curcumin was the most successful in reducing Gli activity and preventing the proliferation of prostate cancer cell line. Based on previous studies, the Hh, and Wnt pathways interact to promote cancer synergistically. There is evidence that drug resistance observed in CSCs may be caused by Hh and Wnt pathways (41). A study examined the effects of curcumin on MCF7 and SUM159 breast CSCs. The study found that curcumin dramatically reduced the expression of key Hh pathway components, including Smo, Gli1, and Gli2. Also, curcumin blocked Wnt/β-catenin, which prevented breast CSCs from increasing (36). Li et al., observed that curcumin reduced the invasion and migration abilities in stable Gli1-overexpressing MDA-MB-231 cells and that vimentin interacted with Gli1 (42). Additionally, an *in vivo* study demonstrated that curcumin downregulated the Hh pathway and restored normal liver pathology in hepatocellular carcinoma-induced rats (43).

### Notch signaling pathway

The Notch pathway defines the interaction between two neighboring cells. The first cell carries a ligand including Jagged-1, Jagged-2, and Delta-like (DLL)-1, -3, and -4, and the second cell has a receptor (Notch-1, -2, -3, -4) that has been programmed to combine with the ligand (44). This interaction initiates the notch pathway and allows the cleavage of the Notch extracellular domain by A Disintegrin and Metalloprotease (ADAM)-10. Thereafter the gamma-secretase complex’s (γ-secretase) cleavage of the Notch intracellular domain (NICD) then translocates into the cell nucleus where it binds to CSL transcription factor. This binding causes the protein known as mastermind-like (MAML) to be recruited in which Notch target genes are transcribed (45). A group of genes involved in angiogenesis, including the VEGF receptors, are regulated by Notch signaling. The ultimate objective of this process is to establish a capillary network that can sustain organ growth, immune system function, and homeostasis (46). Because Notch signaling is elevated in esophageal malignancies, it has been suggested that notch signaling could be a therapeutic target for these diseases (47) Curcumin may inhibit the function of the Notch pathway in cancer by inhibiting Notch pathway activators such as gamma secretases, Notch ligands, or ADAM10. According to a study, in esophageal cancer cells, curcumin inhibits the γ-secretase complex, Notch-1, and its ligand Jagged-1, hence downregulating Notch signaling (43). Further, curcumin downregulated Notch-1 and caused hepatoma cells to undergo apoptosis (48). The modulatory effect of curcumin on the Notch signaling are shown in **Figure 2**. According to recent research, there is a link between curcumin, Notch, and NF-κB. Several downstream target genes are up-regulated because of the expression of several NF-κB subunits in response to Notch. The NF-κB pathway’s activation may have contributed to Notch-induced cell proliferation and apoptosis inhibition (49). In oral squamous cell carcinoma cells, curcumin-mediated reduction of Notch-1 activation also resulted in the downregulation of NF-κB and its target genes, such as Bcl-2, cyclin D1, VEGF, and MMP-9 (50) While in pancreatic cancer cells, curcumin suppressed the Notch signaling pathway, impeded cell development and triggered apoptosis. Thus, inhibiting the activity of NF-κB (51). These data demonstrate a molecular relationship between the Notch-1 signaling system, NF-κB activation, and curcumin’s anticancer properties. Liu et al. demonstrated that increasing doses of curcumin led to greater inhibition of SMMC-7721 hepatoma cells in culture, with corresponding reductions in NOTCH-1 mRNA and protein expression (48). Additionally, the study conducted by Zhang et al., demonstrated that Notch-P53 signaling pathway was down regulated in the presence of curcumin in multiple myeloma cells in Mouse myeloma P3X63Ag8 cells (52)

### JAK/STAT3 signaling pathway

Janus Kinase (JAK)/Signal transducer and activator of the transcription 3 (STAT3) signaling pathway is a cascade of reactions among proteins within the cell that undergo cell growth, proliferation, differentiation, and cell death (53). Considerable evidence demonstrates that the JAK/STAT3 pathway is the primary signaling pathway upregulated to induce cancer-associated fibroblast (CAF) cells in gastric cancer resulting in the proliferation and metastasis of cancer cells. On the other hand, curcumin down-regulates cell proliferation by upregulating the cytokine and chemokine storm to destroy cancer cells (54). Additionally, the JAK/STAT3 signaling pathway has been extensively studied on retinoblastoma cells and they have shown therapeutic effects by promoting cell apoptosis, via enhancing Bax and cleaved caspases 3/9 levels. Moreover, it inhibits proliferation by activating cyclin D and P21 expression in cells (29, 55). Furthermore, another study conducted on papillary thyroid cancer observed that curcumin inhibited PTC by upregulating matrix metalloprotein and inhibited colony formation (55). Therefore, the blockade of STAT3 by inhibitors is a promising therapeutic agent for tumorigenesis and metastasis in gastric cancer, breast cancer, and thyroid cancer. Curcumin exponentially suppressed the viability, invasion, and migration observed in retinoblastoma cells via the phosphorylation of JAK1, STAT1, and STAT3 (56). However, curcumin remarkably inhibits the NSCLS growth and represses tumor angiogenesis (57). Moreover, curcumin downregulated VEGF, B cell lymphoma-extra-large (Bcl-xL), and cyclin D1 via phosphorylation of JAK and STAT3 in NSCLC. Therefore, curcumin might be a potential STAt3-targeting agent for NSCLC therapy. Curcumin is sought to show anti-angiogenic properties by downregulating MMP-2 and VEGF by inhibiting JAK-2 expression and pSTAT3. Moreover, curcumin demonstrated anti-invasive properties by depleting STAT3 in SCLC (58). The study demonstrates that curcumin has the potential to enhance apoptosis in retinoblastoma cells by reducing the anti-apoptotic protein Bcl-2 and increasing the expression of the pro-apoptotic protein Bax and cleaved-caspase-3/9. Additionally, curcumin shows promise in synergistically enhancing the anti-tumor activity of cisplatin on papillary thyroid cancer (PTC) cells and tumor stem cell-like cells by inhibiting STAT3 activity. This indicates that combining curcumin with chemotherapy drugs may lead to a more effective therapeutic outcome, potentially through the JAK/STAT signaling pathway (55). Furthermore, the study conducted by Liang et al., observed that curcumin could inhibit the viability, migration, and invasion of TPC-1 cells by regulating the miR-301a-3p/STAT3 axis (59).

### NF-kβ pathway

The nuclear factor-kappa B (NF - kB) signaling pathway plays a critical role in promoting cell survival, inflammation, and differentiation (60). Curcumin was found to induce inhibitory action in the NFkB activation on hepatoma cells by inhibiting proteins such as JNK, Cyclin D1 and STAT3 (61). Thus, inducing anti-tumorigenic changes. Furthermore, curcumin was studied to induce the inhibitory action on the NFkB pathways in MDA-MB-231 cells by decreasing the expression of transcription factor IAP-1. Thus, inhibiting the survival and anti-apoptotic properties (62). Moreover, curcumin inhibits the invasion and proliferation of cervical cancer cells via impairment of NFkB pathway (47). It was observed to be a potential chemosensitizer via regulation of the NFkB pathway, for instance, a study conducted by Vinod et al. showed that curcumin suppressed the 5-fluorouracil-activated NFkB pathway in MCF-10A. This effect was mediated through the hindrance of IKK activation, phosphorylation, and degradation. Alongside, other proteins such as COX-2, cyclin D1, Bcl, and Bax (60). Another study analyzed that curcumin reduces the expression of the p65 subunit of NFkB in breast cancer cells, resulting in the downregulation of FABP5, PPARβ/δ, and eventually VEGF-A and PDK1 as the downstream target genes involved in cell proliferation, survival, and angiogenesis (62). Furthermore, a recent study conducted by Qiu et al. found that Curcumin slowed osteoarthritis conditions in mice by reducing the expression of ADAMTs-5 and MMP-13 (31). Moreover, curcumin delays the initiation and progression of non-small lung cancer (58). Additionally, the anti-metastatic effect of curcumin in MDA-MB-231 breast cancer was correlated with the reduction in inflammatory cytokine CXCL1 and CXCL2 mRNA and proteins (173). Moreover, miR181b regulates cancer progression by enhancing the cytokine action of CXCL1 and CXCL2 (63).

### Curcumin modification to enhance bioavailability

Pre-clinical studies revealed that oral administration of 10 to 12g of curcumin ultimately results in less than 50nM present in the increased liver metabolism and short half-life, Therefore, curcumin formulations have been explored to increase low oral bioavailability enhancing the compound anticancer potential in patients via optimization of its absorption and serum concentrations (4). Hence, novel approaches have been implemented to combine curcumin with specific adjuvants and formulations in micelles, liposomes, nanoparticles, and phospholipid complexes (5).

Despite the promising anticancer mechanisms, the efficacy of curcumin is hindered by its low bioavailability. Multiple research studies have reported that low concentrations of curcumin were detected in blood, tumors, or extraintestinal tissues, which may be attributed to poor absorption, rapid metabolism or elimination, and chemical instability (3). For instance, a study reported that the oral administration of curcumin at a dose of 500mg/kg only had a 0.06µg/mL serum concentration after 4 hours indicating a bioavailability of 1% (64). Moreover, Muangnoi et al. observed the levels of curcumin at different time intervals. They exposed Caco-2 cells to 5 µM of curcumin and observed that the amount of curcumin in the bioavailable fraction at 15 min was 0.013 µM, with a gradual increase over time, reaching maximum concentrations of 0.055 µM at 60 min. The remaining amount at 4 h was only 0.031 µM indicating the rapid metabolism of curcumin in cells (65). Additionally, the chemical structure of curcumin results in low solubility at neutral or acidic pH levels. It becomes fully protonated in these conditions and can be hydrolyzed in alkaline conditions, particularly in the intestinal environment (pH 6.8). Furthermore, rapid metabolism and systemic elimination occur through the formation of glucuronides and sulfates by conjugation in the intestine. Studies have shown that free curcumin was undetectable, while curcumin glucuronides and sulfates were highly detected in the serum samples of most subjects who were administered curcumin. This indicates the rapid metabolism of curcumin (2).

Therefore, the preliminary step of pharmaceutical strategies is to improve curcumin solubility, absorption, and delivery of hydrophobic curcumin into the system. To achieve this, researchers have implemented the use of curcumin with various delivery systems such as nanoparticles, micelles, conjugates, liposomes, and phytosomal (5, 66). Studies demonstrated that curcumin-loaded noisome nanoparticles (CM-NP) effectively suppressed the viability, proliferation and migration of GSCs by inducing cell cycle arrest and apoptosis (5). The application of CM-NP demonstrates enhanced efficacy in suppressing tumor growth and reducing the invasiveness of GSCs when compared to curcumin alone. Additionally, it exhibits inhibition of monocyte chemoattractant protein 1 (MCP1). Moreover, rats receiving injections of curcumin-loaded PLGA nanoparticles manifested significantly reduced tumor size after five days, whereas the cohort injected with curcumin alone exhibited no significant alterations (67). In addition, hydrophilic poloxamers including poloxamer 407 (PXM - 407) were employed in the formulation of a binary complex solid dispersion (SD). This formulation notably enhanced the solubility of curcumin to 1.266 ± 0.0242 mg/mL and achieved a dissolution rate of 91.36 ± 0.431% within 30 minutes. Importantly, the amorphous nature of the complex ensured the absence of any chemical modification. Consequently, this led to cell cycle arrest at the G2/M phase, ultimately culminating in cellular apoptosis (68). Another *in vivo* study demonstrated that the combination of antisense-oligonucleotide against miR-21 with curcumin-loaded DP micelle complex reduced the tumor volume more effectively than single therapy curcumin or miR21ASO (69).

Moreover, curcumin liposomes are spherules formed by aggregations of hundreds of phospholipid molecules. They compartmentalize bioactive compounds, offering a drug delivery system that can improve treatment by precisely delivering drugs to the targeted site and maintaining therapeutic drug levels over extended periods (70). For instance, in the study conducted by Wang et al., they observed that curcumin and quinacrine liposomes modified with *p*-aminophenyl-α-D-mannopyranoside were a potential preparation to treat brain glioma cells and brain glioma stem cells by inducing apoptosis. Additionally, *in vitro* results suggest that liposomal curcumin induces similar unconjugated curcumin on human pancreatic carcinoma cell proliferation and nuclear factor kappa-light-chain enhancer of activated B-cell (NF-κB) signaling at equimolar concentrations (16). Liposomal curcumin downregulated the NF-κB pathway by consistently suppressing NF-κB binding to DNA, by decreasing the expression of NF-κB-regulated genes, including cyclooxygenase-2 (COX-2) and interleukin (IL)-8, both implicated in tumor growth and invasiveness and subsequently induced apoptosis. *In vivo*, data demonstrated improved bioavailability: liposomal curcumin suppressed pancreatic carcinoma growth in murine xenograft models and inhibited tumor angiogenesis by decreasing the expression of CD31 (endothelial cell marker), vascular endothelial growth factor (VEGF) and IL-8 (71). Furthermore, curcumin-loaded targeted liposomes cross the BBB two-fold higher than the non-targeted liposomes loaded with curcumin For instance, Curcumin-loaded targeted liposomes exhibit enhanced efficacy in inhibiting GBM tumor growth and improving the survival rate of tumor-bearing mice when compared to free curcumin and non-targeted liposome-loaded curcumin (72). Additionally, curcumin phytosome meriva (CCP) demonstrated to improve curcumin bioavailability, which then helps to activate natural killer cells and mediate the elimination of GBM and GBM stem cells (73, 74).

Curcumin conjugates with piperine (1-piperoyl piperidine) and amino acids decrease the metabolism of curcumin in the liver and the intestinal wall thus enhancing the bioavailability by 20 folds without resulting in any adverse effect *in vivo* and *in vitro* (75). A study conducted with human adenocarcinoma cell line showed that the systemic bioavailability of curcumin was increased to 154% with the use of piperine. When piperine was administered with a curcumin volume of 2000mg/kg (76). The study conducted by Patial et al., observed that when Curcumin -piperin conjugation therapy was administered to hepatocellular induced mice, the number of apoptotic cells, was elevated compared to when Curcumin was administered alone. The high apoptotic activity was enhanced via the downregulation of Bcl-2 activity (77). Additionally, curcumin was also conjugated with glycine, glutamic acid, valine and demethylenated piperic acid. These protein carriers or curcumin prevents the metabolic degradation of curcumin thus enhancing tis bioavailability. Additionally, it is aimed to enhance the pharmacological activities, lower the toxicity, improve target specificity and enhance absorption via peptide transporters (78). For instance, in the study performed by, Panada et al., observed that the synthesized conjugates of Curcumin with 3a-q,5a-k and 6a-k exhibited anti-proliferative properties when treated on MCF7, PC3 cells (79). Furthermore, it was observed that curcumin conjugated to (1,7-bis(4-*O*-l-prolinoyl-3-methoxyphenyl)-1,4,6-heptatriene-5-ol-3-one) showed a potent inhibition on the STAT3 dimerization resulting in downregulating the STAT3 transcription factor resulting in inhibition of cell growth and survival (80). However, the conjugation of curcumin with GnRH, a naturally occurring peptide hormone has shown effects of targeted cancer therapy, for instance, the effect of this hybrid was evaluated with MIA-PaCa-2, BxPC-3 and Pan-1 cells, and it presented with inhibition in pancreatic cancer proliferation by inducing apoptotic cell death mediated by caspases-3 and PARP. Interestingly this conjugate presents enhanced water solubility compared to free curcumin, which allows intravenous administration (81). Curcumin in conjugation with antiandrogens induced antiproliferative indices by inhibiting actin-based pseudopodia formation which highly impacted cell migration and tumor metastasis. The study conducted by, Li et al., observed that curcumin in conjugation with bicalutamide enhanced the growth inhibition of androgen-independent prostate cancer cells via SAPK/JNK and MEK/ERK1/2-mediated NF-kB and MUC1-C signaling pathways by inhibiting subunit p65 (82). Whereas, the synergistic effect of Curcumin with immunoconjugates such an antibody has an immunomodulatory and inhibition of tumor growth by targeting the vascular endothelial growth factors (VEGF) and cytokine such as TNF- α. For instance, the study conducted by Nguyen et al., observed that curcumin conjugated with anti-death receptor 5 (DR5) antibody by regulating the ROS and iNOS levels activated in HScs, and it additionally, suppressed the α-SMA expression in fibroblast suppressing the cancer-associated fibroblast activity in enhancing cancer progression (83).

### Clinical trials

To investigate the effect of curcumin on various cancer types, clinical trials were carried out; however, their numbers were lower, and their outcomes were less consistent than those of preclinical research. In a phase I/II experiment, curcumin was found to be safe and acceptable. Research done on patients with inoperable metastatic colorectal cancer demonstrated the efficacy of given oral curcumin at doses up to 2 g daily along with a 12-cycle chemotherapy regimen consisting of 5-fluorouracil, folinic acid, and oxaliplatin (84). Another phase 1 study used curcumin as chemo preventive agent for colorectal neoplasia. Following an initial screening for colonoscopies, the investigators assessed forty patients and examined rectal biopsy samples after four weeks of curcumin (4 g daily) treatment. This study aimed to employ microarray analysis to ascertain curcumin-modified genes that may serve as biomarkers in subsequent chemoprevention investigations. Tolerability and toxicity will also be assessed in this research (NCT01333917). While subjects with recently diagnosed head and neck cancer participate in an open-label, exploratory biomarker phase 1 trial to evaluate the short-term effects of supplementing with a turmeric extract called curcumin C3 complex^®^. The research revealed that consuming turmeric may prevent the growth of cancer (NCT01160302). To find out if curcumin can prevent tumor-induced inflammation in endometrial cancer patients, a phase II study was conducted. Seven patients used Curcuphyt, a standardized 100 mg curcuminoids root extract from *Curcuma longa*, as a dietary supplement. For two weeks, 2 g/day of curcuphyt capsules were administered. However, Curcuphyt-treated group did not vary from control group in terms of inflammatory biomarkers, frequency of immune cell types, T-cell activation, or cyclooxygenase-2 expression (NCT02017353). In a breast cancer patients treated with chemotherapy, a phase II trial was carried out to compare the activity of NF-κB after curcumin intervention versus placebo. The experimental arm received 500 mg of Meriva^®^, a patented curcumin lecithin delivery method, twice a day. The final results are still not published yet (NCT01740323).

On the other hand, data on the therapeutic effect of curcumin in cancer patients is gathered through a systemic evaluation. According to a study, the researchers discontinued two of their trials due to sample size, and they completed the other study even though they knew that the number of patients enrolled was not as high as what was estimated in the protocol description. Furthermore, it was observed that some clinical studies had minimal bias risk, but others had numerous dropouts, which further reduced the overall number of patients and the outcomes found. Therefore, the reported inconsistent results made it difficult to compare the efficacy of curcumin (85).

Curcumin holds a great deal of promise for treating cancer patients, according to newly available clinical study data. Nevertheless, it is unclear if long-term curcumin treatment will have comparable effects. Furthermore, there is inconsistent evidence that curcumin is helpful in treating hematologic malignancies or solid tumors, whether it is used alone or in combination with other traditional antineoplastic drugs. The limited number of clinical trials, the small sample size, the way curcumin is delivered, and the stage of the tumors could all be contributing factors to these contradictory results.

### Future directions of curcumin as an anticancer agent

The main challenges of using curcumin in the treatment of cancer are due to the poor bioavailability, rapid metabolism, and limited stability. Recent advancements in curcumin Nano formulations, have significantly improved its pharmacokinetic profile, making it a promising candidate for cancer therapy. Nanotechnology-based approaches have enhanced curcumin’s bioavailability and stability by converting it into nanoforms that protect it from degradation, improve solubility, and ensure sustained therapeutic effects. These advanced nanoformulations, such as niosomes and cubosomes, encapsulate both hydrophilic and hydrophobic drugs, reduce toxicity, and protect curcumin from physical, chemical, and biological degradation. Such systems have demonstrated superior drug delivery efficiency compared to traditional carriers like liposomes, and they also support topical application, broadening curcumin’s therapeutic utility. The future of curcumin in cancer treatment lies in combination therapies, where it is used alongside conventional chemotherapy, radiation, or immunotherapy. Curcumin’s ability to downregulate pro-survival signaling pathways, enhance drug sensitivity, and modulate immune responses presents opportunities to amplify the effects of standard cancer treatments while minimizing their side effects. Notably, curcumin’s role in overcoming multidrug resistance (MDR) through the inhibition of drug efflux pumps and modulation of drug resistance pathways underscores its potential as an adjunct in combination regimens. Additionally, curcumin’s ability to modulate epigenetic mechanisms, such as DNA methylation and histone modification, highlights its potential to reverse aberrant gene expression in cancer cells. The exploration of curcumin as an epigenetic modulator, particularly when combined with other epigenetic drugs, could provide new strategies to overcome resistance and enhance treatment efficacy. Curcumin’s immunomodulatory effects, including its ability to enhance the activity of immune cells and augment the efficacy of immune checkpoint inhibitors, further expand its therapeutic scope. These properties make curcumin an attractive candidate for integration into immunotherapy protocols, especially in cancers resistant to conventional treatments.

However, despite the promising advances in curcumin research, significant gaps remain in understanding the complete therapeutic profile of nanocurcumin, including optimal dosages, long-term safety, and potential toxic effects. Further studies are needed to fully map its therapeutic potential, explore novel targets and pathways, and develop robust combination therapies that leverage curcumin’s diverse mechanisms of action.

## Conclusion

Curcumin, a common culinary spice, has demonstrated a wide range of benefits in the management and prevention of numerous illnesses. It has been shown to inhibit the growth, proliferation, invasion, metastasis, and angiogenesis of tumor cells, as well as induce apoptosis and overcome treatment resistance. Clinical trials across various cancer types support curcumin’s potential as a significant cancer treatment. However, its poor water solubility and low bioavailability limit its therapeutic effectiveness in clinical settings. Ongoing research aims to address these challenges and enhance curcumin’s therapeutic benefits. Notably, the development of nano-curcumin formulations has improved its anticancer properties. Future advancements in cancer prevention and treatment are likely to increasingly focus on optimizing curcumin’s application.
